# Highly effective NK cells are associated with good prognosis in patients with metastatic prostate cancer

**DOI:** 10.18632/oncotarget.3965

**Published:** 2015-04-29

**Authors:** Christine Pasero, Gwenaëlle Gravis, Samuel Granjeaud, Mathilde Guerin, Jeanne Thomassin-Piana, Palma Rocchi, Naji Salem, Jochen Walz, Alessandro Moretta, Daniel Olive

**Affiliations:** ^1^ Centre de Recherche en Cancérologie de Marseille, Inserm, Marseille, France; ^2^ Aix Marseille Université, Marseille, France; ^3^ Institut Paoli-Calmettes, Marseille, France; ^4^ Dipartmento di Medicina Sperimentale (D.I.ME.S.), Università di Genova, Genova, Italy

**Keywords:** natural killer cells, prostate cancer, metastases, survival, castration resistance

## Abstract

Clinical outcome of patients with metastatic prostate cancer (mPC) at diagnosis is heterogeneous and unpredictable; thus alternative treatments such as immunotherapy are investigated. We retrospectively analyzed natural killer (NK) cells by flow cytometry in peripheral blood from 39 mPC patients, with 5 year-follow-up, and their correlation with time to castration resistance (TCR) and overall survival (OS). In parallel, NK functionality was carried out against prostate tumor cell lines, analyzed for the expression of NK cell ligands, to identify the receptors involved in PC recognition. NK cells from patients with longer TCR and OS displayed high expression of activating receptors and high cytotoxicity. The activating receptors NKp30 and NKp46 were the most obvious predictive markers of OS and TCR in a larger cohort of mPC patients (OS: *p*= 0.0018 and 0.0009; TCR: *p*= 0.007 and < 0.0001 respectively, log-rank test). Importantly, blocking experiments revealed that NKp46, along with NKG2D and DNAM-1 and, to a lesser extent NKp30, were involved in prostate tumor recognition by NK cells. These results identify NK cells as potential predictive biomarkers to stratify patients who are likely to have longer castration response, and pave the way to explore therapies aimed at enhancing NK cells in mPC patients.

## INTRODUCTION

Prostate cancer (PC) is the most common invasive cancer and the second and third leading cause of cancer-related death among men in the U.S. and Europe respectively [1]. Localized tumors can be cured by surgical resection or radiotherapy. For metastatic castrate-sensitive PC (mCSPC) the recommended treatment is castration [2]. However castration resistance inevitably develops for all patients, thus resulting in a need for chemotherapeutic drugs and new treatments for metastatic castrate-resistant disease (mCRPC). Immunotherapy has emerged for over a decade as an alternative therapeutic approach for metastatic patients. The first currently only FDA-approved vaccine is a DC-based vaccine known as Sipuleucel-T (Provenge; Dendreon Seattle, WA, US) which increases survival for mCRPC [3]. Several other immunotherapies for advanced PC have progressed to phase II or phase III clinical trials : the GVAX-PCa (GM-CSF immunotherapy for cancer; Biosante Inc) cell-based cancer vaccine [4, 5], or the Ipilimumab (Yervoy, BMS Princeton, NJ), fully human anti-Cytotoxic T Lymphocyte Antigen-4 (CTLA-4) monoclonal antibody, alone or in combination with radiotherapy [6, 7].

Metastatic PC at the time of diagnosis now represents less than 5 % of PC cases [8]. The median survival of a man with metastases at the time of diagnosis has improved during the last decade from 30 to 49 months due to the PSA (prostate specific antigen) screening, which led to early detection of the disease, and because of new treatments at the time of CRPC [9]. Some prognostic factors have been determined for mPC: bone localizations (axial vs appendicular), visceral localizations, Gleason score ≥ 8, performance status and PSA ≥ 65 ng/ml [10]. Despite these important advances, mPC at the time of diagnosis is still highly heterogeneous and unpredictable, with some patients long responders to sequential treatments, and others non responders. The challenge is now to improve risk stratification tools for this subgroup.

Natural killer (NK) cells belong to innate immune system and exert effector functions such as cytotoxic activity and cytokine production in antiviral and antitumor responses [11]. Human NK cells are defined as CD3-CD56+ cells and represent 5% to 20% of circulating lymphocytes. According to membrane densities of CD56 and CD16, which mediates the antibody-dependent-cellular cytotoxicity (ADCC), NK cells are classified into CD56^dim^CD16+ (90-95% of NK cells) and CD56^bright^CD16− (5-10%) subsets [12]. CD56^bright^ cells are considered immature, rapidly proliferate, produce high levels of cytokines and have the ability to progressively differentiate into CD56^dim^ cells. In contrast, CD56^dim^ cells exert high cytotoxic potential. The effective function of NK cells depends on an intricate balance between activating and inhibitory receptors which are able to bind ligands present on target cells. Inhibitory receptors include the killer immunoglobulin-like receptors (KIR), CD94/NKG2A, and ILT2/CD85j [13-17]. The main activating receptors of NK cells are the natural cytotoxicity receptors (NCRs: NKp46, NKp30, NKp44), NKG2D and DNAM-1 (DNAX accessory molecule-1) [18, 19]. A hallmark of NK cell activation is degranulation, leading to CD107 externalization and release of lytic granule contents (perforin and granzyme) onto the surface of the target cell [20].

A 11-year follow-up pioneering study in human population reported that a low degree of NK cell cytotoxicity was correlated with increased cancer risk [21]. Since then, the prognostic value of NK cells has been explored : a low expression of NKp30 and NKp46 was shown to strictly correlate with enhanced progression of the disease in acute myeloid leukemia [22] and chronic lymphocytic leukemia [23]. There are now accumulating evidences for the role of NK cells in solid tumors; reduced expression of NCRs was associated with different forms of cancer such as melanoma [24-26], cervical cancer [27], breast cancer [28, 29], lung cancer [30-32], renal cell carcinoma [33] and gastrointestinal tumors (GIST) [34, 35]. However NK cells have been very scarcely studied in prostate cancer, excepted immunohistochemical (IHC) observations showing that the ligands for NKp30 and NKp46 are expressed on primary tumors and not on benign prostate hyperplasia [36]. A high count of CD56+ NK cells in prostate tumors after androgen deprivation therapy was associated with a good prognosis, and there was an inverse correlation between the density of CD56+ NK cells and seminal vesicle invasion [37]. Thus, NK cell activity may be a prognosis factor in PC patients.

Here, we analyze NK cell markers monitored *ex-vivo* in peripheral blood of patients with metastases at PC diagnosis and their correlation with clinical outcomes, *i.e.* time to castration resistance and overall survival. We also explore *in-vitro* the recognition mechanisms of prostate tumor cells by NK cells.

## RESULTS

### Patient characteristics

**Figure 1:**
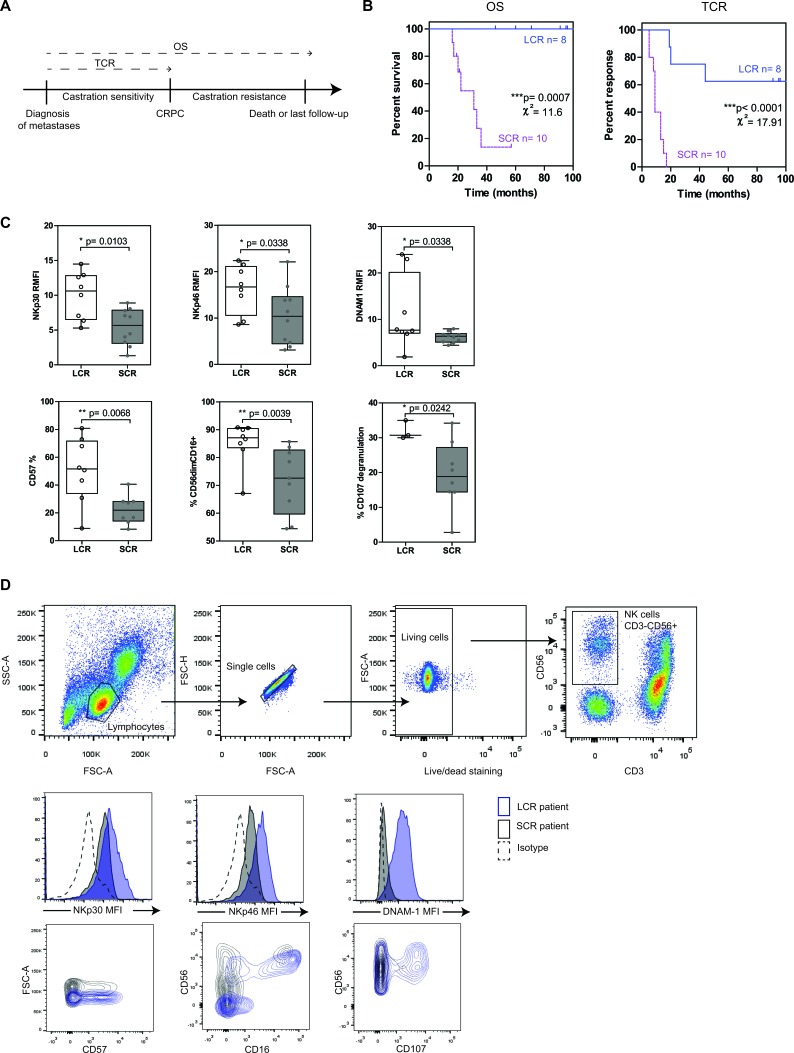
NK cells from mPC patients with longer survival and response to castration have strong cytotoxic potential mPC patients sampled within 2 months after diagnosis of metastases and without treatment at sample (*n* = 18) were stratified into two groups, according to the time to castration resistance with an 18-months cutoff value: LCR (long castration response, *n* = 8) and SCR (short castration response, *n* = 10) patients. **A.** Clinical endpoints analyzed in this study: overall survival (OS) and time to castration resistance (TCR). **B.** Kaplan-Meier curves of OS and TCR. Blue solid line, LCR patients; pink dashed line, SCR patients. The relative differences in survival and response distribution (χ²) and *p* values were determined by log-rank statistics. **C.** The expression of NK cell markers on peripheral NK cells was analyzed by flow cytometry in LCR (white plots) and SCR (grey plots) patients sampled at diagnosis. The y axis shows the MFI ratio or the percentage of NK cells (CD56+CD3-) positive for each marker depending on uni- or bimodal expression. Data are represented by “box and whisker (min to max; horizontal lines represent mean values)” graphs. P values were obtained using Mann-Whitney test. *p* < 0.05 = *; *p* < 0.01 = **; *p* < 0.001 = ***. **D.** Gating strategy for NK cells (CD56+CD3− among living lymphocytes) and representative histogram or dot plot for each NK cell marker.

We conducted a retrospective study to analyze NK cells from a series of 39 patients with metastases at PC diagnosis including rare cases of patients with long-term survival and time to castration resistance (Table 1). Patients were observed for a median period of 62 months (range, 11 to 212 months). For first statistical analyses, patients were excluded if they were under bisphosphonates or corticosteroids at the time of blood sample. Patients were stratified into two groups according to the time to castration resistance, with an 18-months cutoff value: patients with long castration response (LCR), and patients with short castration response (SCR). The clinical characteristics (detailed for each patient in Supplementary Table 1) are summarized in Table 2. The patients selected for first analyses were sampled within two months after diagnosis (*n* = 18). To note, none of the following potential confounding factors: age at diagnosis, initial PSA, initial Gleason score, number and localization of metastases, were statistically different between LCR and SCR patients, even if the distribution of patients with 4 or more metastases, Gleason score ≥ 8 and initial PSA ≥ 65 ng/ml tended to be higher in the SCR compared to the LCR group. In this study, we analyzed two clinical endpoints: the overall survival (OS), measured from the diagnosis of metastases until the date of death or last follow-up; and the time to castration resistance (TCR), measured from the first day of castration until the date of castration resistance (Figure 1A).

**Table 1:** Cohort Among patients with metastases at PC diagnosis (n= 39), patients under treatment at the time of blood sample (corticosteroids or bisphosphonates) were initially excluded. Patients were divided according to the time to castration resistance: LCR pts (long castration response > 18 mo.) and SCR pts (short castration response < 18 mo.). Selected patients for statistical analysis (n=18) have been sampled within two months after diagnosis.

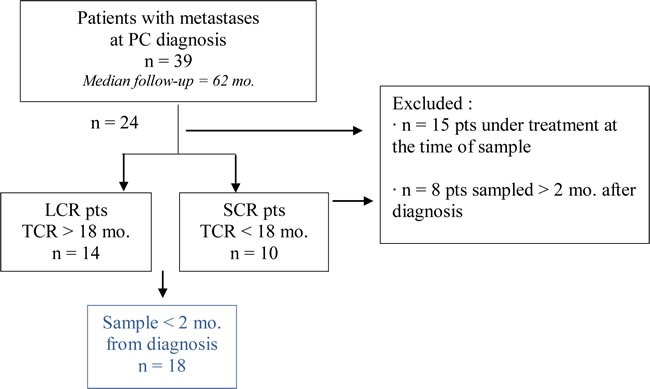

**Table 2:** Clinical characteristics of metastatic PC patients

Clinical parameter	LCR (> 18 mo.)	SCR (< 18 mo.)	p-value
n	14	10	
Sample < 2 mo. from diagnosis	8	10	
Age at diagnosis, yrs (range)	65 (46-81)	68 (53-88)	0.3194*
Initial PSA, ng/ml	290 ± 108	150 ± 53	0.4606*
Initial Gleason Score (Min-Max)	6 - 9	7 - 9	0.1982‡
No. of bone metastases :			
< 4	5 (36%)	2 (20%)	
> 4	4 (29%)	7 (70%)	0.2480‡
None	2(14%)	1 (10%)	
Unknown	3 (21%)	0 (0%)	
Appendicular bone metastases :			
Yes	5 (36%)	6 (60%	
No	7 (50%)	4 (40%)	0.5802‡
Unknown	2 (14%)	0 (0%)	
Axial bone metastases :			
Yes	10 (72%)	9 (90%)	
No	3 (21%)	1 (10%)	0.7688‡
Unknown	1 (7%)	0 (0%)	
Visceral metastases :			
Yes	2 (14%)	4 (40%)	
No	8 (57%)	4 (40%)	0.5507‡
Unknown	4 (29%)	2 (20%)	
Lymph node metastases :			
Yes	2 (14%)	6 (60%)	
No	8 (57%)	3 (30%)	0.0742‡
Unknown	4 (29%)	1 (10%)	
Median OS, mo.	112.1 ± 12.7	27.2 ± 3.9	< 0.0001*
Median TCR, mo.	73.5 ± 13.5	10.3 ± 1.3	< 0.0001*

LCR, long castration response; SCR, short castration response; PSA, prostate specific antigen; OS, overall survival; TCR, time to castration resistance.

Data are indicated as median (range) for age at diagnosis, median (SD) for initial PSA, OS and TCR, n (% of patients) for number and presence of metastases.

* p-value obtained using Mann-Whitney test

‡ p-value obtained using Fisher test

### NK cells from LCR patients display high levels of activating receptors and high functionality

Curves for OS and TCR were thus established for patients sampled at diagnosis (*n* = 18) (Figure 1B). The LCR (*n* = 8) and SCR (*n* = 10) groups were significantly discriminated according to log-rank test (*p* = 0.00007 for OS curves and *p* < 0.0001 for TCR curves). NK cells were isolated from peripheral blood sampled at diagnosis and were characterized *ex-vivo* by flow cytometry for the expression of the major NK cell receptors. Then, univariate analyses using Cox regression model was performed to determine if NK cell markers were significantly associated with OS and TCR. Cox regression analysis (values in Table 3) showed that NKp46, NKp30, DNAM-1, CD56dimCD16+ subset, CD57 and the degranulation marker CD107 were associated with OS (with *p*-values ranging from 0.009 to 0.20). The same markers were found significantly associated with TCR, with superior significant *p*-values (range, 0.002 to 0.10). All these factors have negative z coefficient and thus a positive effect on survival and response to castration.

**Table 3:** Cox regression analyses of NK cell markers on OS and TCR curves

	OS				TCR			
Marker	HR	95% CI	z coeff	p-value	HR	95% CI	z coeff	p-value
NKp46	0.76	0.61 to 0.94	−2.52	0.012	0.83	0.74 to 0.93	−3.05	0.002
% CD56dimCD 16+	0.90	0.84 to 0.97	−2.60	0.009	0.93	0.88 to 0.97	−2.88	0.003
NKp30	0.73	0.54 to 0.98	−2.10	0.035	0.75	0.61 to 0.93	−2.58	0.009
% CD 107	0.89	0.81 to 0.99	−2.18	0.028	0.92	0.86 to 0.99	−2.01	0.044
CD57	0.95	0.89 to I	−1.65	0.098	0.97	0.94 to I	−1.72	0.084
DNAM-I	0.84	0.65 to 1.09	−1.27	0.203	0.82	0.64 to 1.04	−1.61	0.105

OS, overall surviva ; TCR, time to castration resistance; HR, hazard ratio; 95% CI, 95% confidence interval; z coeff, covariate coefficient (indicates the direction and degree of flexing that the predictor has on the survival curve); p-value, significance level

Indeed, we observed that LCR patients (time to castration resistance > 18 mo.) expressed significantly higher levels of NK cell markers than SCR patients (time to castration resistance < 18 mo.) (Figure 1C, 1D): NKp30 (*p* = 0.010, median values of RMFI: 10.6 vs. 5.7 respectively), NKp46 (*p* = 0.034, RMFI median values 16.7 vs. 10.4), DNAM-1 (*p* = 0.034, RMFI median values 7.7 vs. 6.3), CD57 (*p* = 0.007, median values of positive cells: 51.6% vs. 21.9%), CD56dimCD16+ (*p* = 0.004, median values 87.1% vs. 72.6%, Mann-Whitney test). Moreover, NK cells from LCR patients degranulated with higher efficiency than NK cells from SCR patients in response to K562 target cell line (*p* = 0.024, median values 30.7% vs. 18.9%,). To note, CD107 degranulation assay was not available for all patients and thus was excluded in further analyses. Other NK cell markers were tested (*i.e*.; CD69, 2B4, ILT2, NKp44, CD94, NKG2D, % of NK cells, CD16) and were not significantly linked with either OS or with TCR (Supplementary Figure 1A). Interestingly, NK cell markers but not T cell markers were significantly associated with longer survival and response to castration. Only CD69 and DNAM-1 were upregulated in T cells from LCR than SCR patients (Supplementary Figure 1B).

We compared the performance of NK cell markers with clinical parameters (Table 4). In this series of mPC patients, NK cell markers were more performant to discriminate LCR and SCR patients according to the time to castration resistance than the clinical parameters (AUC values for ROC curve; NK cell markers: range 0.76-0.87 and clinical parameters: range 0.51-0.76) (Table 4 and the ROC curves are available on Supplementary Figure 2). The Gleason score and the number of bone metastases were the most discriminant clinical parameters. The cut-off values for NK cell markers and clinical parameters were determined from the highest sensitivity value on the ROC curves. Spearman correlations between NK cell markers and clinical parameters are available in Supplementary Table 2. Particularly, NK cell markers were inversely correlated with the number of bone metastases, the presence of visceral metastases, Gleason score; which are poor prognosis factors in PC, and positively associated with the presence of axial bone metastases, which is a good prognosis indicator.

**Table 4:** Comparison of ROC curve values for NK cell markers and clinical parameters

Test	AUC	p-value	Sensitivity (95% CI)	Specificity (95% CI)	Cut-off value
NKp30	0.82	0.020	75%	60%	6.97
NKp46	0.76	0.062	75%	80%	14.35
DNAM-1	0.79	0.041	87.5%	80%	6.81
% CD57	0.87	0.011	87.5%	87.5%	29.5
% CD56dimCD16+	0.87	0.009	87.5%	77.8%	82.35
Gleason score	0.76	0.080	71.4%	77.8%	≥ 8
Initial PSA	0.56	0.624	53.8%	58.3%	≥ 65 ng/ml
No. of bone metastases	0.71	0.143	71.4%	70%	≥ 4
Appendicular bone metastases	0.57	0.594	62.5%	60%	Yes/ No
Axial bone metastases	0.52	0.894	25%	90%	Yes/ No
Visceral metastases	0.57	0.594	62.5%	60%	Yes/ No
Lymph node metastases	0.59	0.505	62.5%	70%	Yes/ No

AUC, area under the curve; PSA, prostate specific antigen.

The cut-off values for NK cell markers and clinical parameters were obtained from the highest sensitivity value. Cut-off values are expressed as RMFI for NKp30, NKp46 and DNAM-1; as percentage of NK cells positive for CD57 and CD56^dim^CD16+.

We show that NK cells from mPC patients with longer response to castration display efficient phenotypic and functional pattern associated with high expression of activating receptors and molecules involved in NK cell maturation and degranulation. NK cell markers are inversely correlated with prognostic factors associated with poor prognosis.

### NKp30^high^ and NKp46^high^ phenotype predict good prognosis

**Figure 2:**
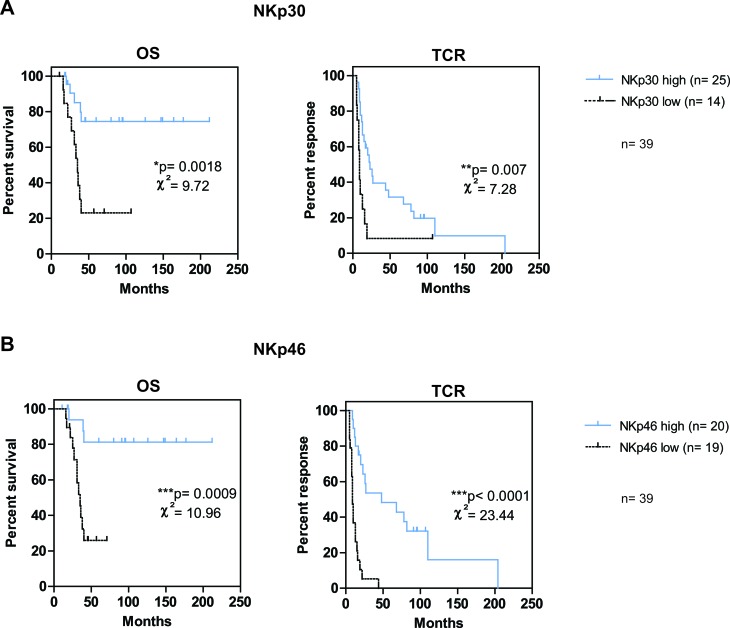
NKp46high and/or NKp30high phenotype is associated with good prognosis in mPC patients Kaplan-Meier curves for OS and TCR were performed on data from the total cohort of 39 mPC patients (*n* = 18 patients used for first statistical analyses and *n* = 21 patients initially excluded because under treatment at sample or sampled at distance from diagnosis). Patients were stratified according to the previously defined cut-off value of NKp30 **A.** and NKp46 **B.** Log-rank statistics were used to compare *high* (solid line) and *low* (dashed line) curves; χ² and *p* values are indicated on each graph with *p* < 0.05 = *; *p* < 0.01 = **; *p* < 0.001 = ***. *n* indicates the number of corresponding patients in each arm.

We determined different subgroups based on NK cell marker expression : patients were stratified into *high* and *low* subgroups according to cut-off values of NK cell markers obtained in Table 4, from the highest sensitivity value on the ROC curves. To obtain a sufficient number of patients for potent statistical analyses, in addition to the 18 patients used for first statistical analyses, we added patients initially excluded because under treatment at the time of blood sample (*n* = 15) or sampled at distance from diagnosis of metastases (*n* = 6). Kaplan-Meier curves for OS and TCR revealed that NKp30 and NKp46 expression significantly predicted OS and TCR in these mPC patients. We first analyzed patient survival and response to castration according to NKp30 expression (Figure 2A). Probability of survival at 3 years was 85 % for patients with NKp30^high^ and 38 % in patients with NKp30^low^ expression (*p* = 0.0018). Probability of response to castration at 3 years was 39% for patients with NKp30^high^ and 8% for the patients with NKp30^low^ expression (*p* = 0.007). We then analyzed patient survival and response to castration according to NKp46 expression (Figure 2B). The expression of NKp46, *high* or *low,* was also predictive of OS and TCR (3-year OS = 94% vs. 39% respectively; *p* = 0.0009; 3-year TCR = 54% and 5% respectively; *p* < 0.0001). The association of CD57, CD56^dim^CD16+ and CD107 subgroups with survival and response to castration was lower (values on Supplementary Table 3). To note, NKp30 and NKp46 remained highly predictive of OS and TCR if log-rank rest was performed only on the smallest cohort of 21 additional patients (see Supplementary Table 3).

### NK cells markers involved in the NK-mediated lysis of prostate tumor cells

**Figure 3:**
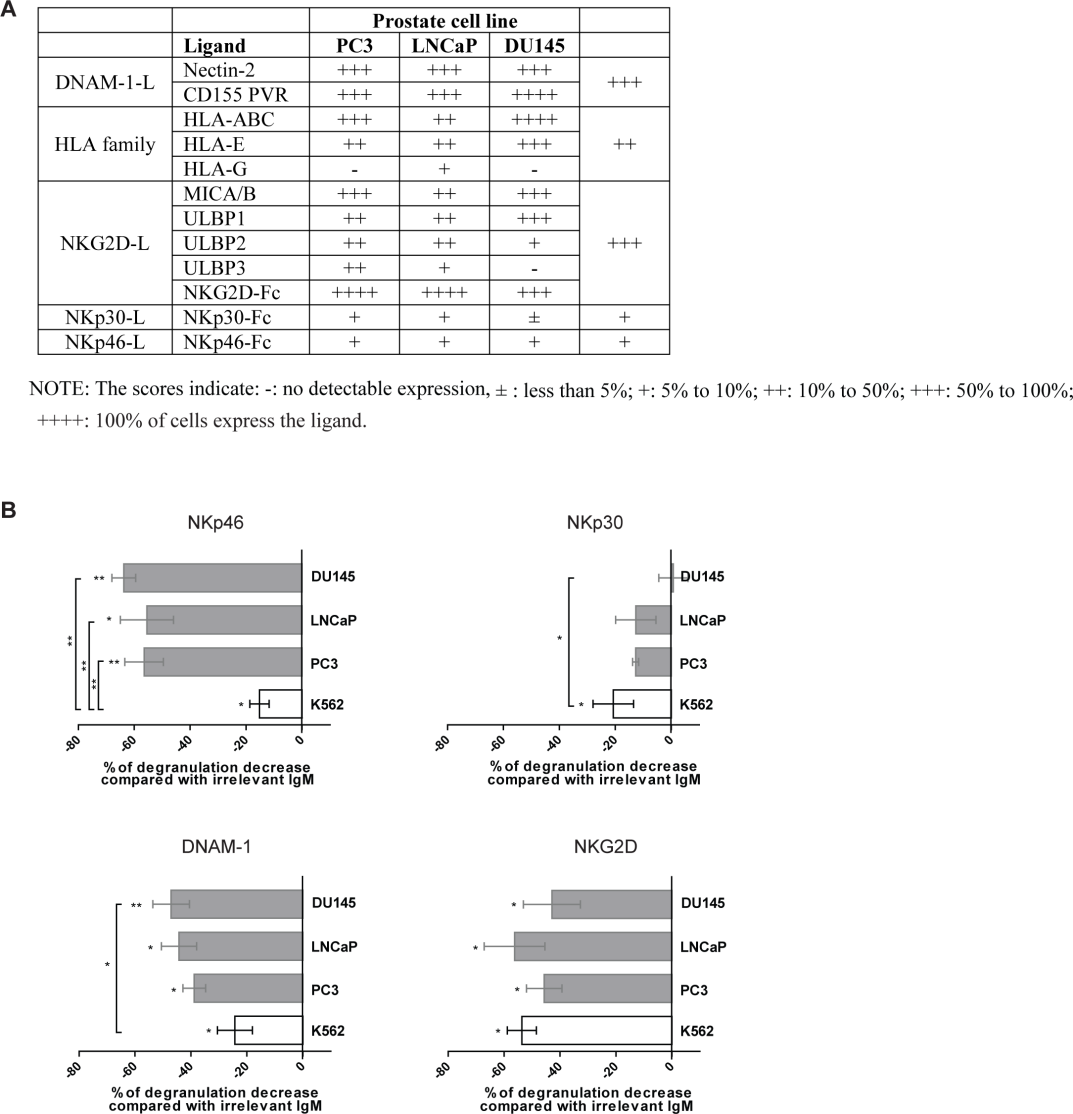
Receptors involved in human prostate cancer cell recognition by NK cells **A.** Expression of NK cell ligands on PC3, DU145 and LNCaP prostate cell lines by flow cytometry. The score indicate: − : no detectable expression; ± : less than 5%; + : 5% to 10%; ++ : 10% to 50%; +++ : 50% to 100%; ++++ : 100% of cells express the ligand. The receptor matching with each ligand is indicated. **B.** PBMCs from healthy donors were activated overnight in IL-2 and IL-15, and used in a 4-hours CD107 degranulation assay against PC3, DU145 or LNCaP prostate tumor cell lines, in the presence of blocking antibodies for NKp46, NKp30, DNAM-1, NKG2D or irrelevant isotype control mAb. E:T = 10:1. The percentage of CD107 degranulation by NK cells was evaluated by flow cytometry. The decreased effect of the respective blocking antibody compared with the irrelevant isotype control mAbs was evaluated with a non-parametric Wilcoxon test. The difference between prostate cell lines and K562 cell line was evaluated with Mann-Whitney test. Results are representative of 4 independent experiments. *p* < 0.05 = *; *p* < 0.01 = **; *p* < 0.001 = ***.

Because NK cell functions are dependent on the expression of ligands at the surface of the target cells, the ligands for NK cell receptors were analyzed by flow cytometry in PC3, LNCaP and DU145 prostate tumor cell lines derived from metastatic prostate adenocarcinoma (Figure 3A). HLA class-I molecules were expressed on all cell lines. The prostate tumor cell lines expressed high levels of MICA/B and ULBP1/2/3, which are ligands for the activating receptor NKG2D, and these observations were confirmed through detection with NKG2D-Fc recombinant protein. Furthermore, Nectin-2 and CD155/PVR which are ligands for the activating adhesion receptor DNAM-1, were highly expressed in PC3, LNCaP and DU145. In contrast, prostate cancer cell lines displayed lower levels of NKp30-L and NKp46-L, even if detection with Fc-molecules has lower binding affinity than specific mAbs.

To determine the receptors involved in prostate tumor cell recognition and their contribution, IL-2/IL-15-activated PBMCs from healthy donors were pre-incubated with blocking antibodies against each NK cell receptor, and then used in CD107 degranulation assay against prostate cell lines or K562 as positive control. The results of the blocking experiments are expressed as percentage of modulation of NK cell degranulation and are summarized in Figure 3B. In accordance with the literature, NKp30, DNAM-1, NKG2D and, to a lesser extent, NKp46 were the receptors involved in K562 cell line recognition by NK cells [38-42]. Indeed, blockage of these receptors inhibited NK cell degranulation against K562. Regarding prostate tumor cell lines, we found that masking NKp46 significantly decreased the degranulation of NK cells against PC3 (56%), LNCaP (55%) and DU145 (64%), with significant higher level than for K562 cell line (*p* < 0.01). Anti-DNAM-1 and anti-NKG2D antibodies also decreased NK cell degranulation against PC3 (39% and 45% respectively), LNCaP (44% and 56%) and DU145 (47% and 43%). Blocking NKp30 decreased modestly NK cell degranulation against PC3 (13%), LNCaP (13%) and DU145 (0.8%) in agreements with the lower expression of NKp30 ligand(s) observed in Figure 3A. The raw data expressed as percentage of CD107ab in presence or absence of blocking mAbs are depicted in Supplementary Figure 3.

Thus, these results identified NKp46, DNAM-1, NKG2D and, to a lesser extent, NKp30 as the receptors regulating the recognition of prostate tumor cells by activated NK cells.

## DISCUSSION

Patients with metastatic prostate cancer at the time of diagnosis have a heterogeneous and unpredictable prognosis, with some patients long responders to sequential treatments, and others non responders. We provide original findings about the correlation of peripheral NK cell phenotype and clinical outcomes in these patients. NK cells from patients with longer survival and time to castration resistance display high expression of NK cell receptors, notably NKp46 and NKp30, which we identify here as involved in prostate tumor cell recognition by NK cells. Hence, the functional experiments corroborate the *ex vivo* data on the identification of the NK cell receptors involved on the control of metastatic PC. Peripheral NK cells from LCR patients display high expression of molecules involved in NK cell cytotoxicity (NKp30, NKp46), costimulation (DNAM-1), maturation (CD57) and degranulation (CD107). To note, higher levels of CD16 in LCR patients suggest a high ability to perform ADCC functions; and this could be exploited to improve the efficacy of therapeutic Ab-mediated effects.

Previous studies in the literature indicate that prostate cancer cells express ligands that could drive NK cell activation. Indeed, exosomes produced by human PC cells express ligands for NKG2D on their surface and induced down-regulation of NKG2D on NK cells [43]. Here, we screened a large panel of NK cell ligands on prostate cancer cell lines and we identified which NK cell receptors are involved in PC recognition. The prostate tumor cells express ligands for the major activating NK cell receptors, in particular high levels of ligands for DNAM-1 and NKG2D, underlying the role of innate immunity in tumor control. We identified NKp46 as the crucial receptor involved in prostate cancer recognition by normal NK cells, along with DNAM-1 and NKG2D, and NKp30 to a lesser extent. Such a role for NKp46 was previously suggested when Arnon et al. showed by IHC that primary human prostate tumors and not benign prostate hyperplasia expressed ligands for NKp46 and NKp30 [36]. Interestingly, our observations also emphasize that the NK cell receptors involved in NK cell antitumor immunity differ according to the tumor type: in breast cancer the main receptors involved in NK cell cytotoxicity are DNAM-1 and NKG2D [29] ; whereas in melanoma NKp30, NKp44 and NKG2D are the receptors that trigger NK cell functions [24]. The recognition tightly depends on the repertoire of ligands expressed by the tumor cells, but also the level of receptors expressed on NK cells. Thus, downregulation of the ligand might be an escape mechanism of the tumor to evade NK cell cytotoxicity, and inversely our results suggest that upregulation of the NK cell receptors- notably NKp46, in some long-responders patients, could favor tumor control in prostate cancer.

Which are the mechanisms that could explain the heterogeneity of NK cell markers observed in metastatic PC patients? NKp46 is frequently deregulated [27, 28] and involved in tumor editing [44]. NKp30 recognizes the tumor antigens B7-H6 and BAG6 (also known as BAT3) but only B7-H6 has been involved in tumor clearance due to its restricted expression on malignant cell lines [38]. Several mechanisms could explain the downregulation of NCRs in some patients: chronic ligands exposure as it has been described for DNAM-1 or NKG2D [29, 45]; or cytokines released by the tumor, such as TGF-β1 which has been shown to downregulate NKp30 and NKG2D [46, 47]. Screening of immunosuppressive factors such as TGF-β1 in the plasma of metastatic PC patients will further be investigated. Recently, elevated levels of cytokines involved in inflammation (IL-8, TNF-α, MCP-1) have been associated with poorer PC outcome [48]. In line with these results, impaired NK cell activity analyzed through IFN-γ levels has been recently associated with prostate cancer progression [49]. Together with our study, these observations strengthen the importance of immune context in PC.

We show that NKp30 and NKp46 are the most predictive markers for OS and TCR in a larger cohort that included patients under treatment or sampled at distance from diagnosis. Interestingly, NKp30 and NKp46 were also the receptors associated with increased survival in AML patients [22]. NKp46 was shown to control melanoma metastases [50] and tumor growth of lymphoma tumors [51] in mouse models and our study emphasizes its role in controlling human metastatic prostate cancer. Distinct NKp30 splice variants have been characterized, demonstrating opposite functional effects. The predominant expression of the immunosuppressive NKp30c isoform was associated with poor prognosis in GIST patients [35]. This could provide additional information to explore whether the relative expression of the different isoforms of NKp30 and/or NKp46 is associated with prostate cancer outcome.

What is the relevance of NK cell markers compared to current prognostic factors? New prognostic factors for metastatic PC have been identified: visceral metastases represent negative prognostic factors and are associated with aggressive disease [52]; in contrast, axial bone metastases are associated with better survival than appendicular metastases [53]. Here, NKp30 and NKp46 meet these emerging prognostic factors: inverse correlation with poor prognostic factor such as visceral metastases and positive correlation with good prognosis factor such as axial bone metastases. To note, the cut-off values that we obtained for clinical parameters were concordant with those described previously in the literature [10].

The NK cell infiltrate is often associated with a lower risk of relapse and/or longer survival: NK cell infiltrate was associated with regression in melanocytic lesions [54], predicted progression-free survival in localized GIST [34], and was associated with a good prognosis in prostate tumors after castration [37]. The NKp30 splice variants determined the prognosis of patients with GIST [35]. Interestingly, we show here that the prognostic value of NK cells is suitable in peripheral blood even in a solid tumor; therefore NK cell markers could be screened on blood samples, easily accessible for routine diagnosis, thus erasing the difficulty to access intratumoral lymphocytes. This suggests that peripheral NK cells may reflect the intratumoral phenotype and would represent tumor-induced subsets as already reported in breast cancer [55]. Together, these data strongly encourage the validation of immune biomarkers for optimal stratification of patients with solid tumors.

The correlation of efficient NK cells in patients with longer time to castration resistance and survival is a strong argument for their involvement in prostate cancer. Thus, our findings strongly suggest that enhancing NK cell efficiency could provide a promising immunotherapeutic approach for metastatic PC; and this work pave the way for further prospective and validation studies. Furthermore, screening for NK cell profiles in peripheral blood provides a non-invasive, easily measurable prognostic biological parameter, and could therefore be useful to improve risk stratification and to design better tailored treatment strategies in metastatic PC patients.

## PATIENTS AND METHODS

### Patients characteristics

Patients with metastatic disease at the time of PC diagnosis were recruited in the period from December 1995 to January 2012 in a comprehensive cancer center (Institut Paoli-Calmettes, Marseille, France). The project was reviewed by the internal review board of Institut Paoli-Calmettes and the study was approved by a central national ethics committee. All patients provided written informed consent. 39 patients with metastatic PC at diagnosis were recruited during this period. To ensure an unbiased analysis, patients were excluded if they were under chemotherapy or radiotherapy at the time of blood sample because of possible effects on immune cells. Patients were stratified into two groups according to the time to castration resistance, with an 18-months cutoff value: patients with long castration response (LCR, median: 73.5 months (mo.) and median OS 112.1 mo.), and patients with short castration response (SCR, median = 10.3 mo. and median OS 27.2 mo.).

The following clinical variables were retrospectively collected: age, initial PSA, initial Gleason Score, number and localization of bone metastases (axial and appendicular), presence of visceral (lung and/or liver) and lymph node metastases, serum levels of: alkaline phosphatase (ALP), lactate dehydrogenase (LDH), hemoglobin, performance status, previous treatments and treatments at the day of blood sample, follow-up (diagnosis of metastases, start of castration, castration resistance diagnosis, death or last follow-up). Clinical data where more than 30% of values per group were missing were excluded from further analyses (ALP, LDH, hemoglobin and performance status). Clinical characteristics are summarized in Table 2, and detailed along with the treatments received for each patient during their follow-up in Supplementary Table 1.

### PBMC isolation from blood samples

Peripheral blood mononuclear cells (PBMC) were purified from blood samples by Lymphoprep (Lymphocyte separation medium, Eurobio, France) density gradient centrifugation. The cells from peripheral blood were washed and resuspended in RPMI (Roswell Park Memorial Institute, Gibco Life Technologies) medium and then frozen before phenotype and functional analysis.

### Flow cytometry analyses

Peripheral NK cells were characterized ex vivo by multicolor flow cytometry analyses. Thawing cells were incubated with conjugated antibodies or isotypic controls for 30 minutes at 4°C, washed and extemporaneously analyzed on LSRFortessa cytometer (BD Biosciences). NK cells (gated on CD3-CD56+ in lymphocyte FSC/SSC parameters, followed by elimination of the doublets based on FSC-A/FSC-H parameters and removal of dead cells using a cell viability marker, Live/Dead Aquadead kit (Invitrogen, Life Technologies, Grand Island, NY)) were labeled with PE (Phycoerythrin)- conjugated antihuman mAbs: NKp44 (clone Z231), NKp30 (clone Z-25), NKp46 (clone BAB281), 2B4 (clone C1.7), NKG2D (clone ON72), CD94 (clone HP-3B1), CD69 (clone TP1.55.3), ILT2 (CD85j, clone HP-F1; Beckman Coulter, Miami, FL), DNAM-1 (CD226, clone F22; BD Pharmingen, San Jose, CA), CD57 (clone HCD57; Biolegend, San Diego, CA), and allophycocyanin-Cy7 (APC-Cy7)- CD16 (clone 3G8). Data analysis was done using FACSDiva (BD Biosciences) or FlowJo (TreeStar Inc.) programs. The phenotypic data in the paper are represented as the percentage of positive cells or the expression level (RMFI for ratio MFI; ratio between the specific and control isotype staining) depending on the marker.

Prostate tumor cell lines were labeled with HLA-ABC-FITC (clone B9.12.1, Beckman Coulter), HLA-E-PE (clone 3D12HLA-E, eBioscience), HLA-G-FITC (clone MEM-G/9, Abcam), MICA/B-PE (clone 6D4, BD Pharmingen), ULBP1-PE (clone 170818), ULBP2-PE (clone 165903), ULBP3-PE (clone 166510, R&D Systems), Nectin-2-PE (clone R2.477.1, Beckman Coulter), CD155-PE (clone SKII.4, Biolegend). NKp30-Fc, NKG2D-Fc and NKp46-Fc (R&D Systems) chimeric proteins were used to detect ligands for NKp30, NKG2D and NKp46 respectively.

### Cell lines

PC3, DU145 and LNCaP cell lines are derived from metastatic prostate cancer samples, castrate-resistant for PC3 and DU145; and castrate-sensitive for LNCaP. Cell lines were purchased from American Type Culture Collection (ATCC). PC3 was cultured in Dulbecco's modified Eagle's medium (DMEM, Gibco Life Technologies) supplemented with 10% fetal calf serum (FCS, Lonza), and DU145 and LNCaP were cultured in RPMI supplemented with 10% FCS. K562 cell line is derived from human leukemia cell line (ATCC) and was cultured in RPMI supplemented with 10% FCS.

### CD107 degranulation assay

To evaluate the functionality of NK cells from PC patients, PBMCs from patients were activated overnight in IL-2 (100 U/ml, Chrion) and IL-15 (20 ng/ml; Miltenyi Biotec), and incubated with K562 cell line at effector : target (E:T) ratio of 10:1 during 4 hours at 37°C, with monensin (Golgi Stop, BD Biosciences), FITC-conjugated anti-CD107a (LAMP1) and FITC-conjugated anti-CD107b (LAMP2) mAbs. Cells were then washed in PBS and stained for 30 minutes at 4°C with PerCP-Cy5.5-conjugated anti-CD3 and PC7-conjugated anti-CD56 antibodies. The percentage of CD3-CD56+ NK cells positive for CD107 was analyzed on a LSRFortessa cytometer (BD Biosciences).

For blocking experiments to determine which receptors are involved in prostate tumor cell recognition by NK cells, PBMCs from healthy donors were preincubated 30 min with blocking mAbs or saturating concentrations of appropriate isotype controls. Blocking mAbs directed against NK cell receptors were produced in Pr Moretta's laboratory: anti-NKp30 (F252), anti-NKp46 (KL247), anti-DNAM (F5), anti-NKG2D (ON72). Then, prostate tumor cell lines or K562 cell line as positive control were added at effector : target (E:T) ratio of 10:1 followed by the same protocol as above.

### Methodology and statistical analyses

All statistical analyses were performed using Prism software (GraphPad), except Cox regression with R 3.01 software. Correlations between patient groups and clinical features were analyzed using the Mann-Whitney test or the Fisher's exact test (variable with two groups). Overall survival (OS) was measured from the date of diagnosis of metastases until the date of death or the last follow-up. Time to castration resistance (TCR) was measured from the first day of castration until the date of castration resistance. OS and TCR were estimated using the Kaplan-Meier method and curves were compared with the log-rank test. Univariate Cox proportional hazard regressions were performed with R software to analyze the effect of several risk factors on OS and TCR curves. Receiver operating characteristic (ROC) curve analysis was used to assess the discrimination performance of biological and clinical parameters, and to identify the optimal cutoff value. The Mann-Whitney test was used to compare two groups of data (LCR vs SCR patients) and the Wilcoxon test was used to compare paired data in functional experiments with blocking antibodies. Statistical significance was accepted at the 5% level with no adjustment for multiple testing.

## SUPPLEMENTARY MATERIAL FIGURES AND TABLES



## Abbreviations

CSPC: castrate-sensitive prostate cancer
CRPC: castrate-resistant prostate cancer
GS: gleason score
mPC: metastatic prostate cancer
NCRs: natural cytotoxicity receptors
NK: natural killer
OS: overall survival
PSA: prostate specific antigen
TCR: time to castration resistance
