# Supplementary material for: Highly effective NK cells are associated with good prognosis in patients with metastatic prostate cancer
Source: Oncotarget. 2015 Apr 29;6(16):14360–73. doi: 10.18632/oncotarget.3965 (PMC4546472; doi:10.18632/oncotarget.3965)
Supplement: Supplementary file 1 [file oncotarget-06-14360-s001.pdf]

Highly effective NK cells are associated with good prognosis in patients with metastatic prostate cancer

Supplementary Material

Supplementary Figure 1a. NK cell markers not statistically discriminant between LCR and SCR patients.

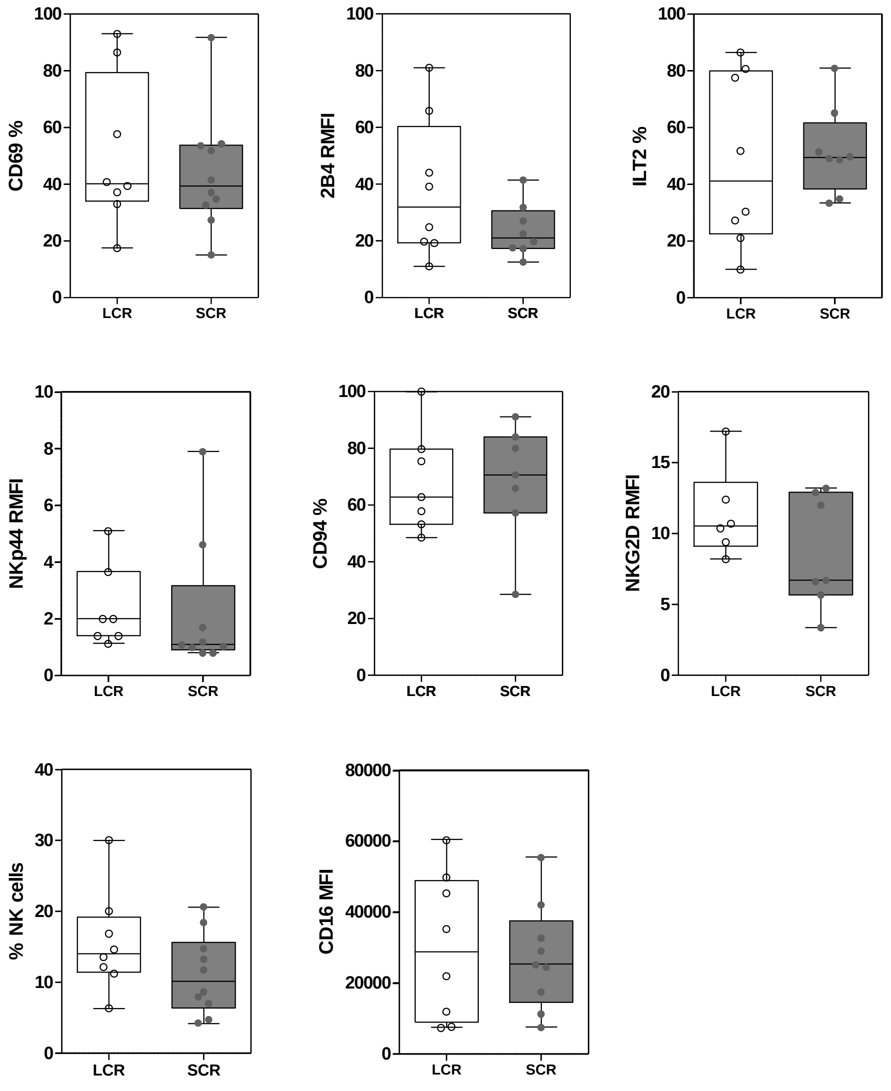

Supplementary Figure 1A. NK cell markers not statistically discriminant between LCR and SCR patients (CD69, 2B4, ILT2, NKp44, CD94, NKG2D, % of NK cells and CD16). The expression of NK cell markers on peripheral NK cells was analyzed by flow cytometry in LCR (white plots) and SCR (grey plots) patients sampled at diagnosis. Data are represented by “box and whisker (min to max; horizontal lines represent mean values)” graphs. n= 18 mPC patients.

Supplementary Figure 1b. Expression of the markers on CD3+ and CD8+ cells.

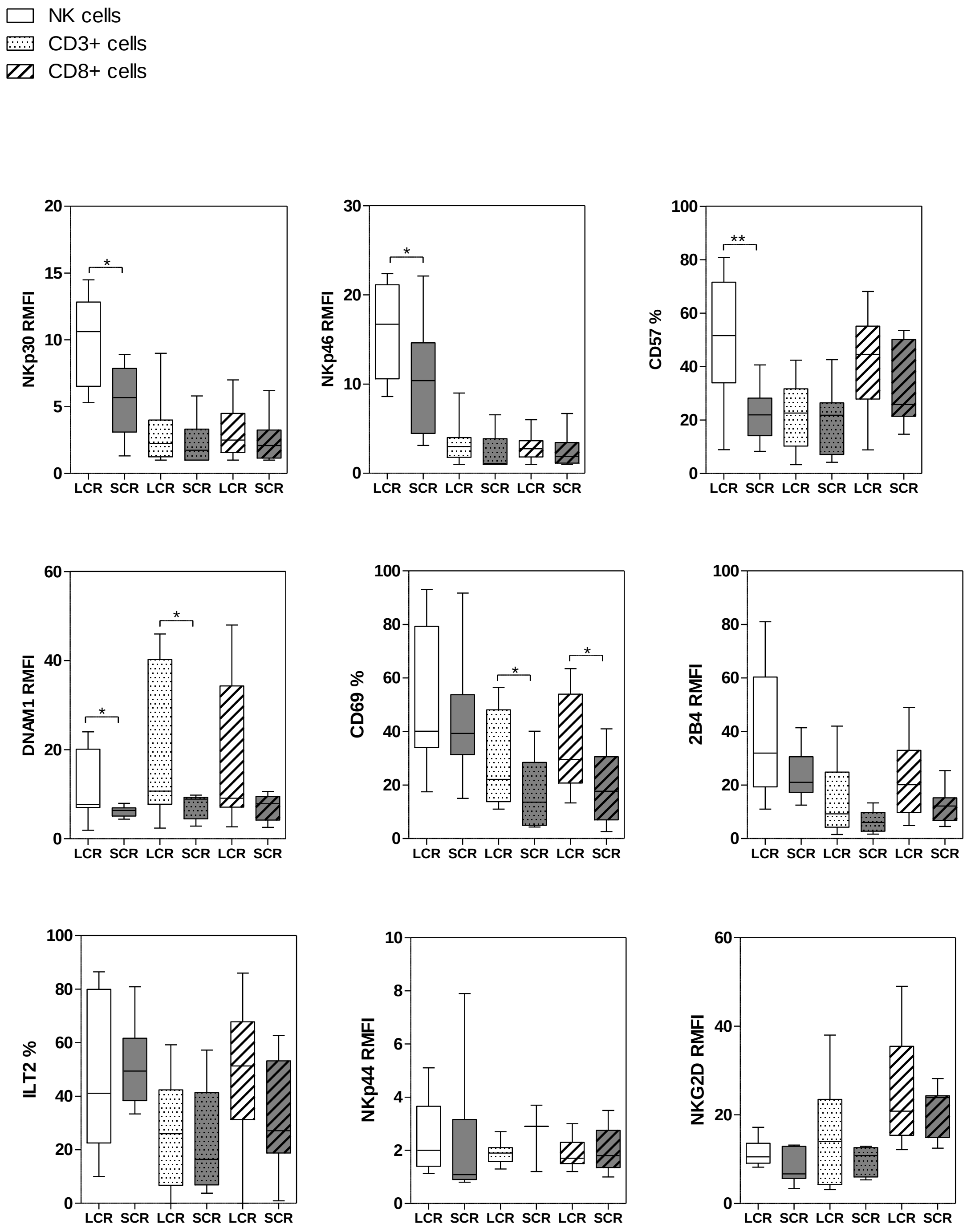

Supplementary Figure 1B. Expression of the markers on NK cells, CD3+ cells (spotted box plot) and CD8+ cells (dashed box plots). Data are represented by "box and whisker (min to max; horizontal lines represent mean values)" graphs. n= 18 mPC patients.

Supplementary Figure 2. ROC curves for NK cell markers.

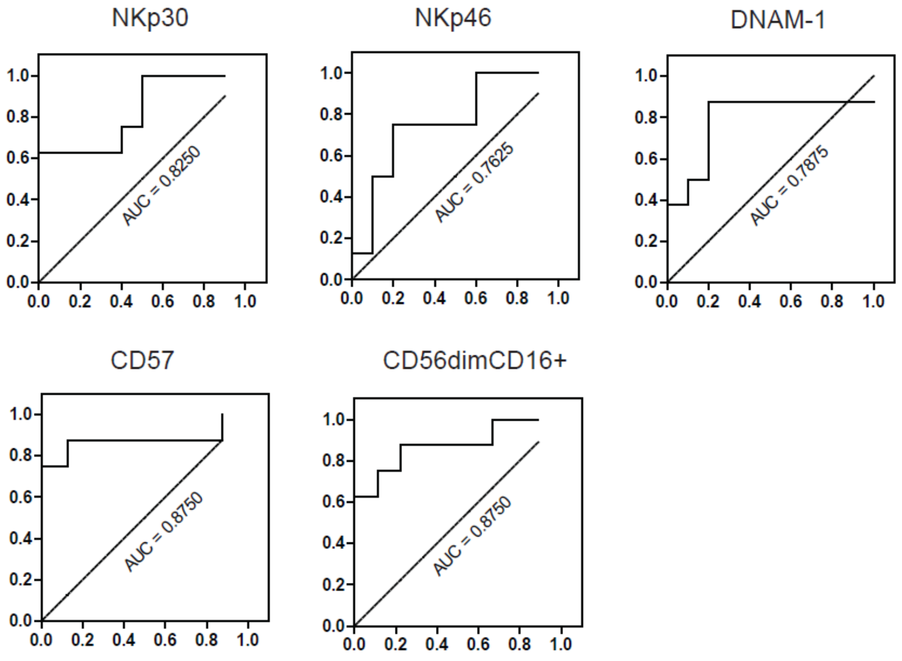

Supplementary Figure 2. ROC curves for NK cell markers. ROC curves used to establish AUC (area under the curve) values, % of sensitivity and specificity, cut-off values indicated in the Table 4. n =18 mPC patients.

Supplementary Figure 3. NK cell degranulation against prostate cell lines in presence of blocking antibodies.

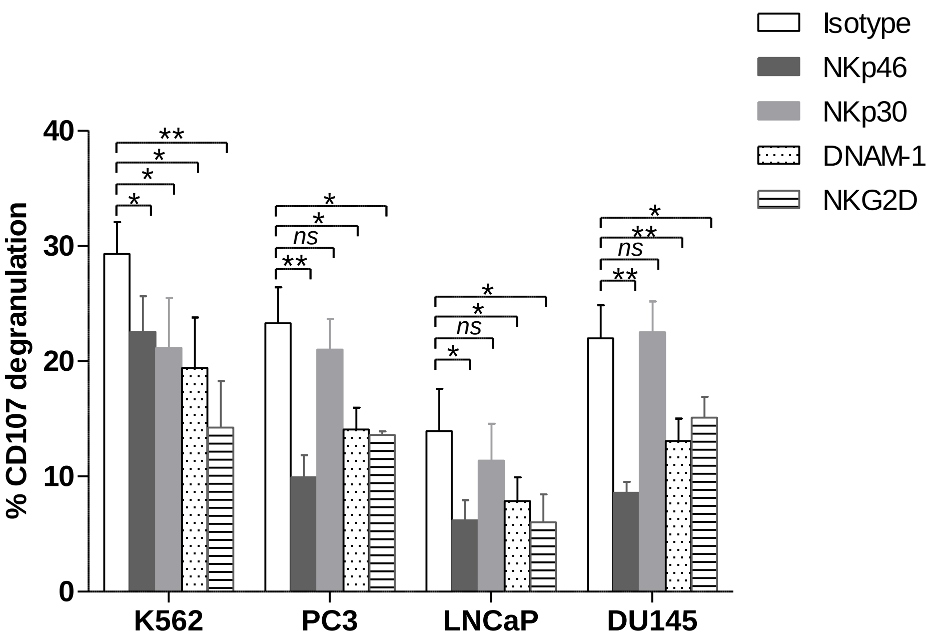

Supplementary Figure 3. NK cell degranulation against prostate cell lines in presence of blocking antibodies. PBMCs from healthy donors were activated overnight in IL-2 and IL-15, and used in a 4-hours CD107 degranulation assay against PC3, DU145 or LNCaP prostate tumor cell lines, in the presence of blocking antibodies for NKp46, NKp30, DNAM-1, NKG2D or irrelevant isotype control mAb. E:T = 10:1. The percentage of CD107 degranulation by NK cells was evaluated by flow cytometry. The decreased effect of the respective blocking antibody compared with the irrelevant isotype control mAbs was evaluated with a non-parametric Wilcoxon test. Results are representative of 4 independent experiments. $p<0.05$=*; $p<0.01$=**; $p<0.001$=***.

Supplementary Table 1. Characteristics and treatments of the patients.

Patient	GS	PSA (ng/ml)	Age (yrs)	Treatment received	Group
p1	6	3.33	76	-	LCR (CSPC at sampling)
p2	7	31.2	46	Txt, Abi, Bisphosphonates	
p3	9	206	81	-	
p4	9	17.89	65	Txt, Bisphosphonates	
p5	6	165	65	-	
p6	6	343	72	-	
p7	7	63	54	Bisphosphonates	
p8	na	na	66	Abi, Bisphosphonates	
p9	8	12.7	61	Txt, Bisphosphonates	LCR (CRPC at sampling)
p10	na	na	68	-	
p11	na	14	69	CT*, Bisphosphonates	
p12	7	700	72	Txt*	
p13	na	1000	70	Abi	
p14	7	927	50	Bisphosphonates	
p15	7	168	54	Txt, Abi, Bisphosphonates	SCR (CSPC at sampling)
p16	8	38	53	Txt, Bisphosphonates	
p17	8	120	83	-	
p18	9	340	55	Txt, Bisphosphonates	
p19	na	114	76	Txt, Abi	
p20	9	18	63	-	
p21	7	94	88	Abi	
p22	9	65	53	Txt, Abi, CT, Bisphosphonates	
p23	9	5.26	76	Bisphosphonates	
p24	9	542	75	-	

Abbreviations: LCR, long castration response; SCR, short castration response; GS, Gleason Score; PSA, prostate specific antigen; Txt, Taxotere; Abi, Abiraterone; CT, chemotherapy; na, not available

Note: all patients received castration treatment

* indicates treatment received before blood sample, other treatments were received after sample.

GS and PSA were based on biopsy specimens at the time of initial diagnosis prior to castration

Supplementary Table 2. Correlation of NK cell markers with clinical parameters.

	**NKp30**		**NKp46**		**DNAM-1**		**CD57**		**CD56dimCD16+**		**CD107**	
**Variable**	**r**	**p**	**r**	**p**	**r**	**p**	**r**	**p**	**r**	**p**	**r**	**p**
Gleason Score	-0.02	0.47	-0.29	0.14	-0.38	0.07	-0.65	***0.004***	-0.58	***0.01***	-0.03	0.46
Initial PSA	0.03	0.45	-0.01	0.48	0.01	0.48	-0.44	***0.05***	-0.13	0.32	0.17	0.31
No. of bone metastases	-0.15	0.28	-0.15	0.29	-0.64	***0.003***	-0.58	***0.01***	-0.53	***0.02***	-0.14	0.34
App. bone metastases.	-0.19	0.23	-0.24	0.18	-0.55	***0.01***	-0.39	0.08	-0.45	***0.04***	-0.35	0.15
Axial bone metastases	0.28	0.13	0.13	0.31	-0.38	0.07	-0.50	***0.03***	-0.36	0.08	-0.15	0.33
Visceral metastases	-0.42	0.06	-0.05	0.42	-0.31	0.14	-0.19	0.27	-0.20	0.24	-0.04	0.46
LN metastases	-0.41	0.07	0.19	0.25	-0.34	0.12	-0.43	0.08	-0.25	0.21	-0.17	0.33

r indicates the correlation coefficient; p indicates p-value (Spearman correlation).
Statistically significant p values are indicated as italic bold values.
PSA, prostate specific antigen; App, appendicular; LN, lymph node

Supplementary Table 3. Log-rank values for OS and TCR curves.

Log-rank values for OS and TCR curves. Log-rank statistics were used to compare *high* and *low* curves of each marker. “High” and “low” subgroups were based on the cut-off values determined on Table 4. (A) Statistics for the total cohort of 39 mPC patients; (B) Statistics for the additional 21 mPC patients only.

A/ Cohort n= 39 patients

	OS		TCR	
**Marker**	χ^2	**p-value**	χ^2	**p-value**
NKp30	9.72	0.0018**	7.28	0.007**
NKp46	10.96	0.0009***	23.44	<0.0001***
DNAM-1	3.98	0.0461*	2.68	0.1017
% CD57	5.4	0.0201*	6.46	0.011*
% CD56$_{dim}$CD16+	0.70	0.4012	4.58	0.0324*

B/ Cohort n= 21 additional patients

	OS		TCR	
**Marker**	χ^2	**p-value**	χ^2	**p-value**
NKp30	6.11	0.0135*	5.50	0.019*
NKp46	8.50	0.0036**	12.80	0.0003***
DNAM-1	0.002	0.97	0.701	0.40
% CD57	0.24	0.62	0.07	0.79
% CD56$_{dim}$CD16+	2.80	0.09	0.36	0.55
